# Supplementary material for: Onset of Visible Capillary Waves from High-Frequency Acoustic Excitation
Source: Langmuir. 2023 Mar 1;39(10):3699–709. doi: 10.1021/acs.langmuir.2c03403 (PMC10018762; doi:10.1021/acs.langmuir.2c03403)
Supplement: Supplementary file 1 — la2c03403_si_001.pdf [file la2c03403_si_001.pdf]

Supporting Information: Onset of visible capillary waves from high-frequency acoustic excitation

Shuai Zhang,[1,2] Jeremy Orosco,[1] and James Friend*[1,2,3]

[1] *Medically Advanced Devices Laboratory,*
Center for Medical Device Engineering and Biomechanics,
Department of Mechanical and Aerospace Engineering, Jacobs School of Engineering,
University of California San Diego, La Jolla, CA 92093-0411 USA

[2] *Materials Science and Engineering Program, Jacobs School of Engineering,*
University of California San Diego, La Jolla, CA 92093-0411 USA

[3] *Department of Surgery, School of Medicine,*
*University of California San Diego, La Jolla, CA 92093 USA**

INDEX

S.A. Derivation of acoustic streaming equations based on the slow streaming assumption 1

S.B. Simulation results illustrating the static mode of fluid interface deformation from acoustic pressure 2

S.C. An illustrated algorithm for the acoustic pressure present on the fluid interface 4

S.A. DERIVATION OF LINEAR MASS AND MOMENTUM CONSERVATION EQUATIONS BASED ON THE SLOW STREAMING ASSUMPTION

We begin with the Navier-Stokes equations as follows, conserving mass and momentum:

$$\frac{\partial \rho}{\partial t} + \nabla \cdot (\rho u) = 0 \tag{1a}$$

$$\rho \frac{\partial u}{\partial t} + \rho (u \cdot \nabla) u = -\nabla p + \mu \nabla^2 u + (\mu_B + \frac{\mu}{3}) \nabla \nabla \cdot u) \tag{1b}$$

* jfriend@ucsd.edu

The terms in equations (1) can be decomposed into three contributions:

$$
\begin{cases}
u = u_0 + \epsilon u_1 + \epsilon^2 u_2 + \mathcal{O}[\epsilon^3] & \text{(2a)} \\
p = p_0 + \epsilon p_1 + \epsilon^2 p_2 + \mathcal{O}[\epsilon^3] & \text{(2b)} \\
\rho = \rho_0 + \epsilon \rho_1 + \epsilon^2 \rho_2 + \mathcal{O}[\epsilon^3]; & \text{(2c)}
\end{cases}
$$

u_0, p_0, and ρ_0 are hydrostatic terms and those with subscripts 1 and 2 refer to first and second-order perturbations. The variable ϵ is a Mach number, defined here as the ratio of fluid velocity to the speed of sound ($\epsilon = u_1/c_0$). Since the fluid velocity is small in this system, $\epsilon \ll 1$. Introducing the expansions (2)(a-c) into eqns. (1)(a,b) and grouping in terms of ϵ, the resulting equations can be separated into three parts according to the zeroth, first, and second order components of the acoustic perturbation. The first-order acoustic perturbation expression represents the behavior of the linear acoustic waves in the fluid. Since the dimensions of the droplet are small and the fluid velocity is likewise small, the Reynolds number in this case is also a small value. So the equations can be simplified as follows:

$$\frac{\partial \rho_1}{\partial t} + \rho_0(\nabla \cdot u_1) = 0, \tag{3}$$

$$\rho_0 \frac{\partial u_1}{\partial t} = -\nabla p_1. \tag{4}$$

S.B. STATIC MODE SIMULATION RESULTS

Two simulation prediction of the static deformation of the fluid interface are provided in Fig.S1 for (a) water from 1.1 nm acoustic waves, and (b) a 90%–10%wt glycerol-water solution from 3.3 nm acoustic waves. After the application of 1.1 nm amplitude 6.6 MHz ultrasound to the water droplet, it produced a sudden increase in height and thereafter a static response, akin to past observations [1]. Upon the application of 3.3 nm amplitude 6.6 MHz ultrasound to the glycerol-water combination, the sudden increase in height was followed by capillary waves that were strongly damped. The horizontal axis in these results is reported as *number of states simulated* as the simulation is quasi-static. This axis can be transformed to a time-based prediction using the capillary wave dispersion relation as described in the previous subsection.

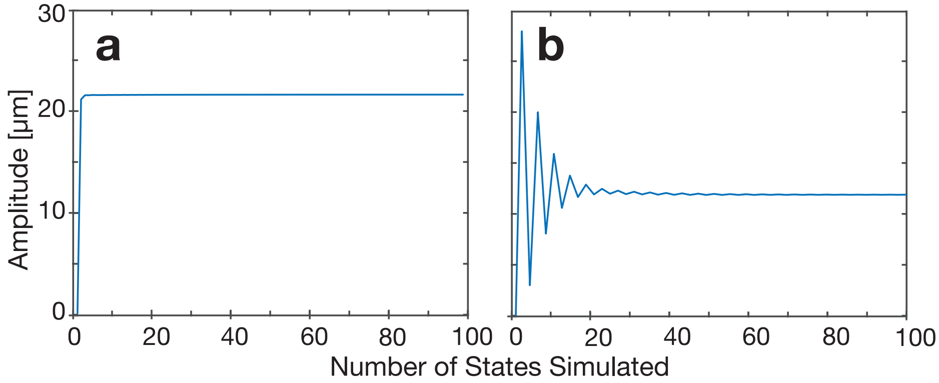

FIG. S1. Simulated deformation of the fluid interface in response to 6.6 MHz acoustic waves. A sudden displacement and static response were induced in (a) the water sessile droplet from 1.1 nm acoustic waves, while the (b) 90%-10%wt glycerol water droplet surface exhibited both the sudden displacement and the appearance of strongly damped capillary waves from 3.3 nm acoustic waves.

S.C. ALGORITHM USED IN THE PRESSURE-INTERFACE FEEDBACK MODEL

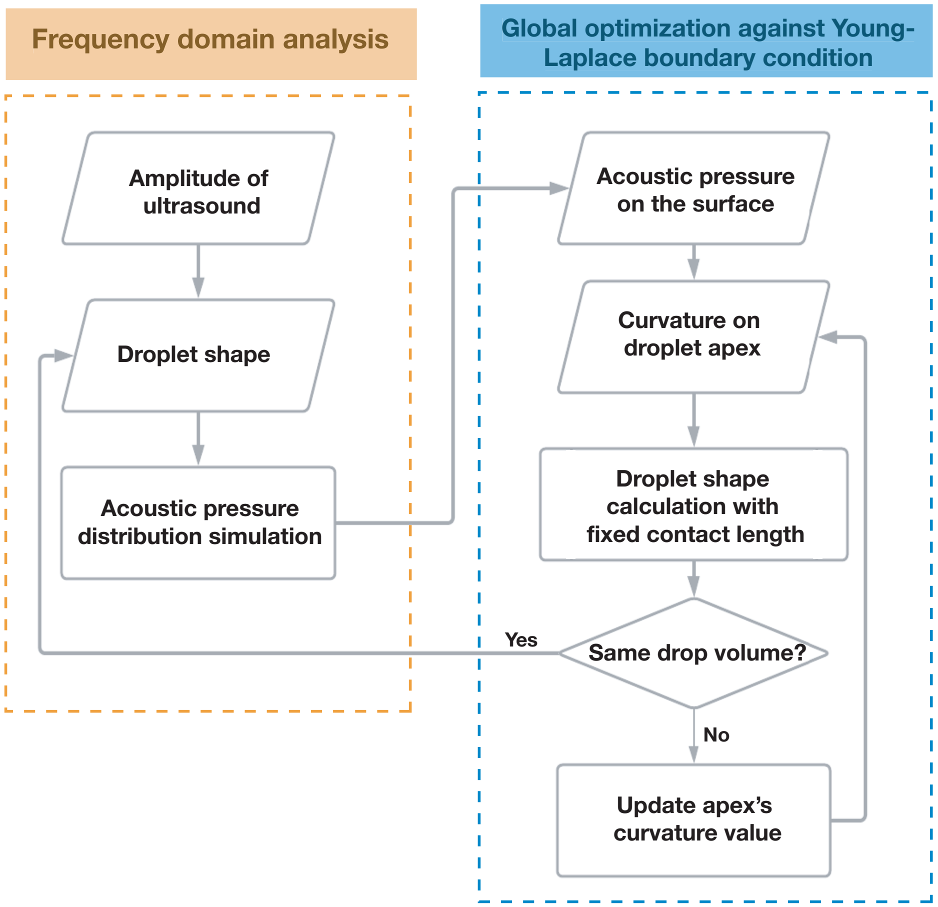

FIG. S2. The algorithm to simulate the shape of the droplet and the acoustic pressure distribution.

REFERENCES

[1] Manor, O.; Dentry, M.; Friend, J. R.; Yeo, L. Y. Substrate dependent drop deformation and wetting under high frequency vibration. *Soft Matter* **2011**. *7*, 7976–7979.
